# Christmas at the Hospitals

**Published:** 1888-01-21

**Authors:** 


### CHRISTMAS AT THE HOSPITALS.

(BY OUR COMMISSIONER AND CORRESPONDENTS.)

### ST. THOMAS'S.

OWING to Christmas Day falling on Sunday the entertainments at this hospital were spread over the week that followed. On the day itself the patients had an old English dinner of roast beef and plum pudding, and it was very pleasing to the resident officers of the hospital, who visited each ward during the dinner-hour, to hear the patients express such gratitude for the kindness and attention they had received during their stay there. Besides this feast the patients had during the evening the pleasure of hearing a number of carols from the choir of Nightingale probationers. During the succeeding evening there were entertainments and festive tea-parties in the various wards. Mr. Toole and other friends kindly came in to assist. On the 11th of January those patients who were able to leave the wards were present at an entertainment in one of the operating theatres, made for the nonce the home of a brighter scene than usual. In this Mr. Alfred Caldicott, Miss Grace Arnold, and Mr. Walter Fletcher took part, assisted by an amateur orchestra. The second part of the entertainment consisted of a performance by the "St. Thomas's Hospital Negro Minstrel Troupe," which met with great applause. The matron, Miss Jones, the steward, Mr. Fred Walker, and the other officials, must have been much gratified by the appreciation their efforts to amuse the patients met with.

### BRITISH HOME FOR INCURABLES.

A VERY successful Christmas entertainment took place at the above institution at Clapham on Tuesday evening, the 10th inst., under the direction of Mr. Charles E. Combe, assisted by the Misses M. and N. Daniel, Miss Edith Evans, Mr. W. Barlow, Mr. N. Davis, Mr. W. Miles, Mr. E. Hollingsworth, Mr. C. Appleton, and Mr. Stanley Hazell. The entertainment concluded with a performance of *The Spitalfields Weaver*. Owing to the very severe weather, many of the patients were unable to be present; but a very pleasant evening was spent, and afforded great gratification to the poor sufferers who were able to leave their rooms, and to the staff generally. The secretary, Mr R. G. Salmond, one of the ablest and most successful of secretaries, at the conclusion of the entertainment, made a few remarks, thanking those ladies and gentlemen who had kindly come down on such a foggy evening for so kind a purpose.

### SOUTH DEVON HOSPITAL.

THE festivities at the South Devon and East Cornwall Hospital extended over two days. On Sunday the annual dinner to the patients took place, when they were all regaled with turkey and plum pudding, and were very well looked after. As an extra treat the visitors were allowed to stay two hours instead of the usual one in the afternoon, and the inmates thus had the opportunity of a long chat with their friends. The wards were decorated with banners and evergreens and looked very nice and bright; the patients, those who were able to rise, sitting round a real Christmas fire, and the others, in their beds, looked very comfortable and happy. By the generosity of the gentlemen of the committee and other friends they each received a useful present on Christmas morning, consisting of an article of wearing apparel, a book, or something equally likely to be appreciated. The children were, of course, not forgotten, but received dolls and other toys.

